# Pathology of the neurovascular unit in leukodystrophies

**DOI:** 10.1186/s40478-021-01206-6

**Published:** 2021-06-03

**Authors:** Parand Zarekiani, Marjolein Breur, Nicole I. Wolf, Helga E. de Vries, Marjo S. van der Knaap, Marianna Bugiani

**Affiliations:** 1 Department of Pathology, Amsterdam UMC, Vrije Universiteit Amsterdam and Amsterdam Neuroscience, de Boelelaan 1117, 1081HV Amsterdam, The Netherlands; 2 Amsterdam Leukodystrophy Center, Amsterdam UMC, Amsterdam, The Netherlands; 3 Department of Molecular Cell Biology and Immunology, Amsterdam UMC, Vrije Universiteit Amsterdam and Amsterdam Neuroscience, Amsterdam, The Netherlands; 4 Department of Child Neurology, Emma Children’s Hospital, Amsterdam UMC, Vrije Universiteit Amsterdam and Amsterdam Neuroscience, Amsterdam, The Netherlands

**Keywords:** Neurovascular unit, Leukodystrophy, White matter, Astrocytes, Endothelium, Pericyte, Microglia, Oligodendrocyte, Myelin

## Abstract

The blood–brain barrier is a dynamic endothelial cell barrier in the brain microvasculature that separates the blood from the brain parenchyma. Specialized brain endothelial cells, astrocytes, neurons, microglia and pericytes together compose the neurovascular unit and interact to maintain blood–brain barrier function. A disturbed brain barrier function is reported in most common neurological disorders and may play a role in disease pathogenesis. However, a comprehensive overview of how the neurovascular unit is affected in a wide range of rare disorders is lacking. Our aim was to provide further insights into the neuropathology of the neurovascular unit in leukodystrophies to unravel its potential pathogenic role in these diseases. Leukodystrophies are monogenic disorders of the white matter due to defects in any of its structural components. Single leukodystrophies are exceedingly rare, and availability of human tissue is unique. Expression of selective neurovascular unit markers such as claudin-5, zona occludens 1, laminin, PDGFRβ, aquaporin-4 and α-dystroglycan was investigated in eight different leukodystrophies using immunohistochemistry. We observed tight junction rearrangements, indicative of endothelial dysfunction, in five out of eight assessed leukodystrophies of different origin and an altered aquaporin-4 distribution in all. Aquaporin-4 redistribution indicates a general astrocytic dysfunction in leukodystrophies, even in those not directly related to astrocytic pathology or without prominent reactive astrogliosis. These findings provide further evidence for dysfunction in the orchestration of the neurovascular unit in leukodystrophies and contribute to a better understanding of the underlying disease mechanism.

## Introduction

Leukodystrophies are monogenic disorders characterized by primarily affected central nervous system (CNS) white matter (WM) regardless of the genetic defect and structural component involved [1]. They are classified following the cell type that drives the degeneration of the CNS WM [2], into diseases of oligodendrocytes and myelin, of astrocytes, of microglia, and of blood vessels. The disease course is often progressive and fatal. For most leukodystrophies, no curative treatment is known.

The neurovascular unit (NVU) refers to a functional cellular complex present in the microvasculature of the CNS, formed by specialized brain endothelial cells (BECs) that form the blood–brain barrier (BBB), of which the barrier properties are maintained through continuous interaction of its surrounding cells, such as astrocytes, microglia, neurons and pericytes. Together, the NVU is responsible for neurovascular coupling (NVC), the mechanism by which local blood supply is adjusted to neuronal demand via changes in vascular intraluminal diameter. Thus, NVU regulates brain nutrient and oxygen supply by controlling cerebral blood flow (CBF). Proper function of the NVU is also essential for maintenance of brain homeostasis by separating the blood from the CNS [3]. The first line of defense is formed by a layer of BECs, which are tightly sealed by tight junction (TJs) proteins like claudins and occludin, together with adherens junctions (AJs) proteins as VE-cadherin. BECs lack fenestrae and have low rates of transcytosis, thereby limiting transcellular and paracellular passage of molecules into the CNS. Nutrients, metabolites and other essential molecules are actively transported across the BECs into the CNS by specific polarized transporters, whereas waste products are actively removed from the CNS by another class of polarized transporters, such as ATP binding cassette (ABC) transporters. The second cell type in the NVU is the pericyte. These cells share the inner basement membrane with BECs and are directly connected to the BECs via peg-socket junctions, which are composed of connexins and N-cadherin [4]. Via these peg-socket junctions, there is a direct exchange of ions, metabolites and other small molecules between pericytes and BECs. Also, pericytes are responsible for dynamic modulation of the vessel diameter, thereby regulating CBF, which can in turn influence the rate of exchange of molecules crossing the BBB. A third crucial component of the NVU is the astrocyte. Within the CNS, astrocytes have different functions, including maintenance of ion-water homeostasis, support of myelination, regulation of glutamate transport and synthesis, enabling synaptic plasticity, control of immune reactions and promotion of neurite outgrowth [5–8]. At the NVU, astrocytes are connected with their endfeet to the outer (glial) basement membrane. These endfeet are specialized and polarized structures containing orthogonal arrays of intramembranous particles, which express high levels of the water channel aquaporin-4 (AQP4), as well as Kir.4.1, an ATP-sensitive potassium channel. These channels are responsible for ion-water homeostasis at the NVU [9–12]. Moreover, astrocyte-derived soluble factors modulate the TJ and transporter expression in the BECs, and thereby regulate NVU function [10]. Astrocytes also regulate the non-cellular component of the NVU, i.e. the basement membranes. All the cells within the NVU contribute to the development of the basement membrane by secreting extra-cellular matrix (ECM) molecules, such as laminins, collagen, fibronectin and dystroglycans, making the basement membrane a highly dynamic structure depending on the input from the surrounding cells. Microglia also participate in the NVU. Microglia are the innate immune cells of the CNS [13, 14].

Involvement of the NVU has been documented in many neurological diseases, with immune-mediated diseases and neurodegenerative polio-encephalopathies being investigated the most. Knowledge about the NVU in other diseases is virtually lacking, amongst these are the leukodystrophies. An involvement of the NVU in leukodystrophies is shown by magnetic resonance imaging (MRI) using contrast agents, that reveals leakage of the BBB in patients. A problem is that administration of contrast agents is not common clinical practice in leukodystrophies and that therefore knowledge of contrast enhancement in leukodystrophies is incomplete. In addition, leakage of contrast agents in MRI highlights only coarse abnormalities and more subtle abnormalities remain unnoticed. This clinical and neuropathological lack of knowledge prompted us to investigate whether the NVU is involved in leukodystrophies of different cellular origin.

## Materials and methods

Frontal lobe tissue samples obtained from the middle portion of the right middle frontal gyrus from 9 leukodystrophy patients and 3 non-neurological controls were obtained at autopsy (within 4 h post-mortem) at the Amsterdam Leukodystrophy Center. The patients or their guardians provided informed consent for the use of the tissue for research purposes. The study was approved by the hospital Medical Ethical Committee and according to the Declaration of Helsinki. The patients’ and controls’ clinical details are provided in Table 1.

**Table 1:** Patients’ and controls’ demographic features

Donor	Age at death (years)	Sex	Clinical diagnosis	Abbreviation	Primary cell type involved
Patient 1	17	F	Aicardi-Goutières syndrome	AGS	Astrocytes
Patient 2	32	M	Alexander disease, adolescent-onset	AxD	Astrocytes
Patient 3	13	F	Alexander disease, infantile-onset	AxD	Astrocytes
Patient 4	75	F	Cathepsin A-related arteriopathy with strokes and leukoencephalopathy	CARASAL	Blood vessels
Patient 5	32	F	Adult-onset leukodystrophy with spheroids and pigmented glia	ALSP	Microglia
Patient 6	36	F	*LARS2*-related leukodystrophy		Astrocytes
Patient 7	5	F	Metachromatic leukodystrophy	MLD	Oligodendrocytes
Patient 8	14	M	Pelizaeus-Merzbacher disease	PMD	Oligodendrocytes
Patient 9	70	M	X-linked adrenoleukodystrophy	X-ALD	Oligodendrocytes
Control 1	27	M	Lymphocytic myocarditis		
Control 2	5	F	Viral pneumonia		
Control 3	12	M	Osteosarcoma		

Formalin-fixed paraffin-embedded frontal WM samples were cut at 5 µm and subsequently deparaffinized in xylene, rehydrated in descending grades of alcohol and washed in PBS. Aldehyde groups were blocked in a glycine solution for 10 min, followed by antigen retrieval in 0.01 M citrate buffer (pH 6.0) in a microwave for 3 min. Sections were cooled down to room temperature and then washed in PBS. Sections were incubated in primary antibodies overnight at room temperature. Primary antibodies targeted Ulex Europaeus Agglutinin I (UEA I, B-1065, 1:1000, Vector Labs), glial fibrillary acidic protein (GFAP, AB5541, 1:500, Merck), AQP4 (AB3594, 1:100, Millipore), α-dystroglycan (05-593, 1:50, Millipore), claudin-5 (34–1600, 1:50, Invitrogen), platelet-derived growth factor-beta (PDGFRβ, 610,113, 1:50, BD Bioscience), and ZO-1 (33–9100, 1:50, Invitrogen). After incubation, sections were rinsed in PBS, incubated with the corresponding secondary antibodies (Alexa fluor 647; S11223, Molecular Probes; Alexa fluor 594 A11005, A11012, Thermo Fisher Scientific; Alexa fluor 488, A11039, Thermo Fisher Scientific) 1 h at room temperature and rinsed with PBS. Tissue autofluorescence was quenched with Sudan Black for 10 min, followed by washing in PBS. Finally, sections were embedded in FluormountG + DAPI (Southern Biotech, Birmingham, Alabama, U.S.) followed by slip covering. A Leica DM5600 (Leica Microsystems BV, Rijswijk, the Netherlands) was used for imaging the sections.

## Results

**Fig. 1:**
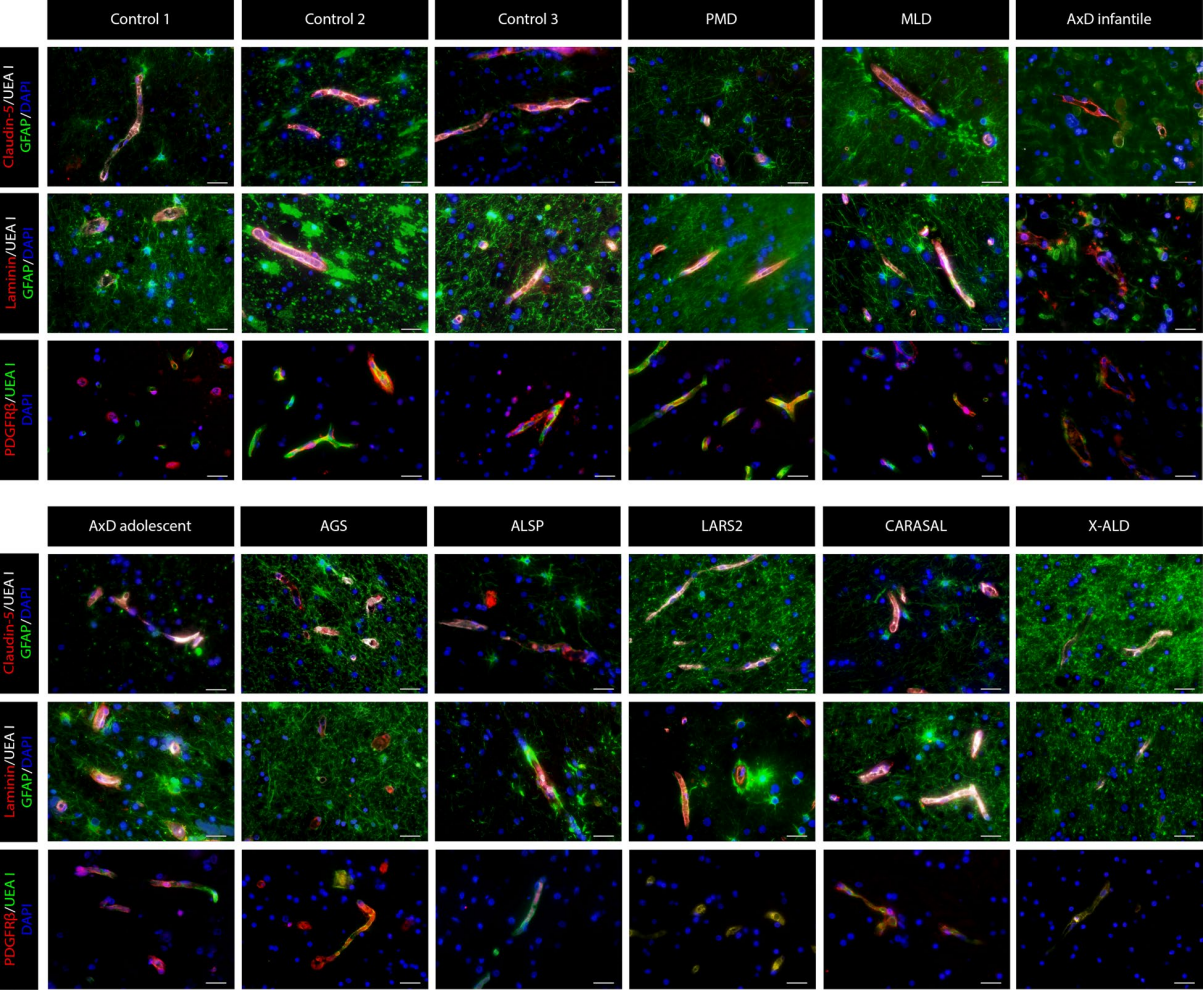
**Claudin-5, laminin and PDGFRβ expression in the frontal WM of non-neurological controls and leukodystrophy patients.** Claudin-5 is expressed in between endothelial cells (UEA I) in all subjects. No peculiarities were seen in its expression in the leukodystrophies. Astrocytes are stained with GFAP and nuclei with DAPI. Laminin in the controls is, as expected, only expressed at the vasculature, where it stains the basal membrane. The expression of laminin is only altered in *LARS2*-related leukodystrophy, where astrocytes show cytoplasmic vesicular laminin immunoreactivity. Astrocytes are stained with GFAP, endothelial cells with UEA I and nuclei with DAPI. PDGFRβ in controls is only expressed in the vasculature, where it stains pericytes. The expression of PDGFRβ is not altered in leukodystrophies. Endothelial cells are stained with UEA I and nuclei with DAPI Scale bar = 25 µm

**Fig. 2:**
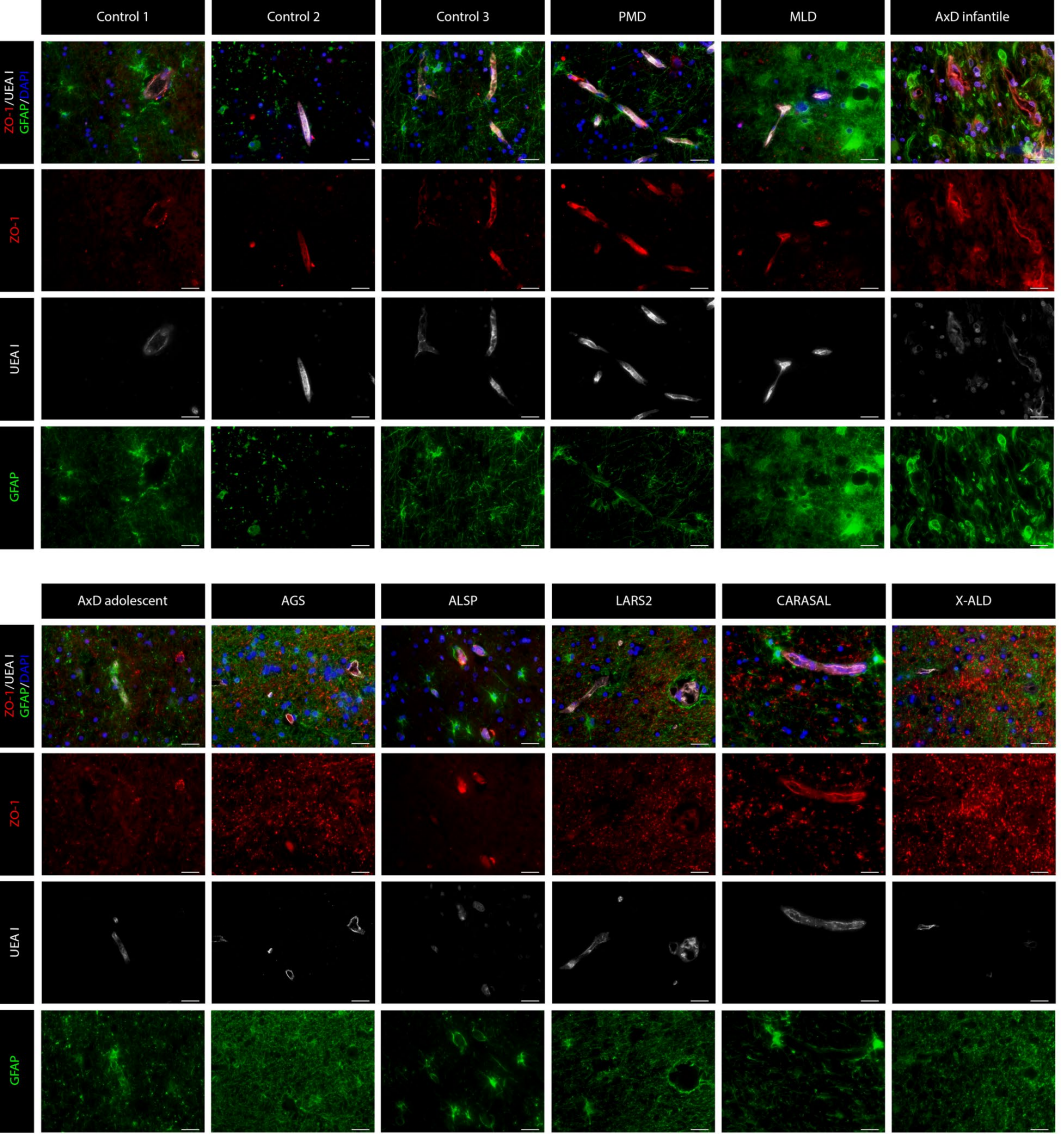
**ZO-1 expression in the frontal WM non-neurological controls and leukodystrophy patients. **ZO-1 is expressed in between the endothelial cells (UEA I) of non-neurological controls. The expression pattern of ZO-1 in PMD, MLD and ALSP is as that observed in controls. By contrast, in AxD (infantile- and adolescent-onset), AGS, *LARS-2*-related leukodystrophy, CARASAL and X-ALD, the expression of ZO-1 is also observed outside the vasculature, where it shows a vesicular pattern in the extracellular matrix. Astrocytes are stained with GFAP (green) and nuclei with DAPI. Scale bar = 25 µm

**Fig. 3:**
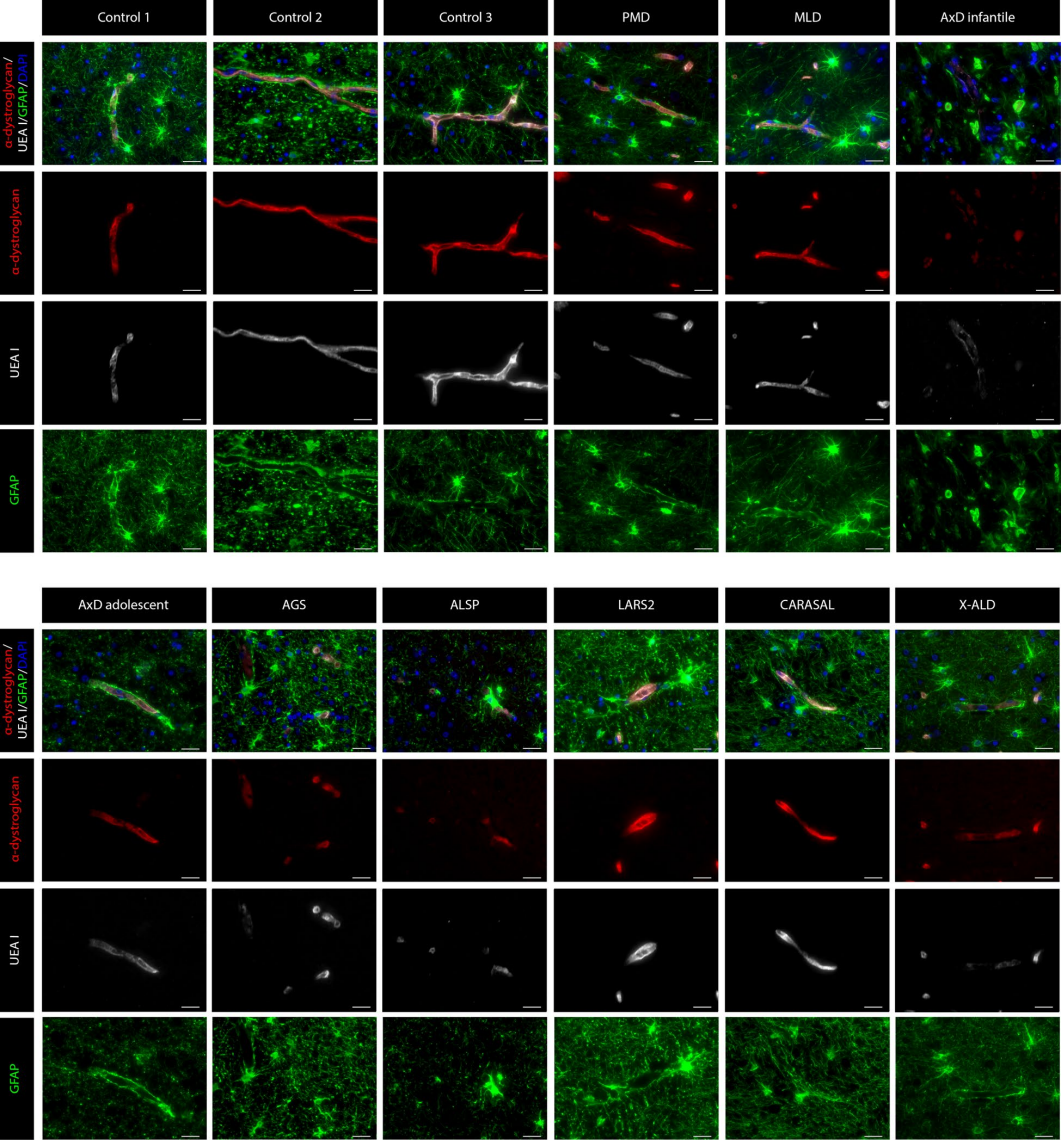
**α-dystroglycan expression in the frontal WM of non-neurological controls and leukodystrophy patients.** α-dystroglycan in controls is expressed at the vasculature, where it stains in between basement membrane and astrocyte endfeet. Its expression is reduced in blood vessels with reduced UEA I expression, including AxD, ALSP, X-ALD and AGS. Astrocytes are stained with GFAP, endothelial cells with UEA I and nuclei with DAPI. Scale bar = 25 µm

**Fig. 4:**
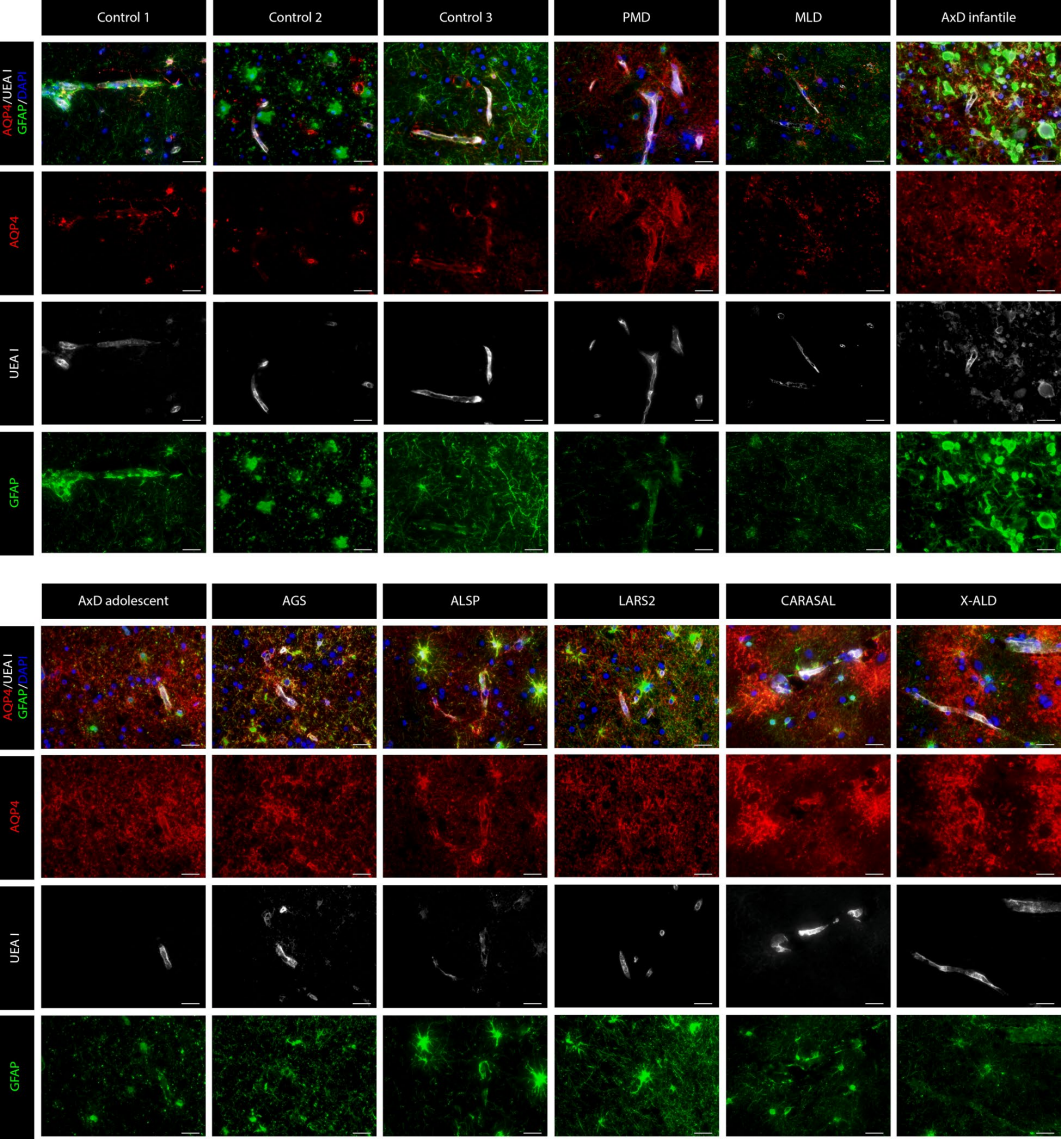
**AQP4 expression in the frontal WM of non-neurological controls and leukodystrophy patients.** In controls, AQP4 is only expressed at the perivascular astrocytic endfeet, as expected. In all assessed leukodystrophies, there is prominent redistribution of AQP4 to the plasma membrane of non-blood vessel related astrocytic cell processes. Astrocytes are stained with GFAP, endothelial cells with UEA I and nuclei with DAPI. Scale bar = 25 µm

Structural changes in the NVU in leukodystrophies were assessed using immunohistochemistry. We chose one or more leukodystrophy patient representative of each disease category, and investigated expression of claudin-5 (Fig. 1), ZO-1 (Fig. 2), laminin (Fig. 1), PDGFRβ (Fig. 1), α-dystroglycan (Fig. 3) and AQP4 (Fig. 4) together with UEA I and/or GFAP. A summary of the results is provided in Table 2.

**Table 2:** Changes in blood–brain barrier and neurovascular unit component in leukodystrophies

Patient	Expression pattern of protein of interest					
	Endothelium		Basement membrane	Pericyte	Astrocyte	
	Claudin-5	ZO-1	Laminin	PDGFRβ	AQP4	α-dystroglycan
AGS	Unchanged	Redistribution	Unchanged	Unchanged	Redistribution	Low expression in vessels with low UEA I expressio
AxD adolescent-onset	Unchanged	Redistribution	Unchanged	Unchanged	Redistribution	Low expression in vessels with low UEA I expression
AxD infantile-onset	Unchanged	Redistribution	Unchanged	Unchanged	Redistribution	Low expression in vessels with low UEA I expression
CARASAL	Unchanged	Redistribution	Unchanged	Unchanged	Redistribution	Unchanged
ALSP	Unchanged	Unchanged	Unchanged	Unchanged	Redistribution	Low expression in vessels with low UEA I expression
*LARS2*-related leukodystrophy	Unchanged	Redistribution	Expression in perivascular astrocytes	Unchanged	Redistribution	Unchanged
MLD	Unchanged	Unchanged	Unchanged	Unchanged	Redistribution	Unchanged
PMD	Unchanged	Unchanged	Unchanged	Unchanged	Redistribution	Unchanged
X-ALD	Unchanged	Redistribution	Unchanged	Unchanged	Redistribution	Low expression in vessels with low UEA I expression

Pelizaeus–Merzbacher disease (PMD) is the prototypic hypomyelinating leukodystrophy. These diseases feature impaired developmental myelination in the CNS [15]. PMD is an X-linked disorder caused by changes in *PLP1,* encoding proteolipid protein 1 (PLP1) and the alternative spliced variant DM20 [16]. PLP1 and DM20 are solely expressed by oligodendrocytes in the CNS and are crucial components of the myelin sheath [17]. In PMD, MRI shows diffuse hypomyelination and increasing WM atrophy [18–20]. No contrast enhancement has been reported. Pathology shows lack of myelin and oligodendrocytes due to altered PLP1 levels that activate the unfolded protein response, leading to apoptosis [21–23]. Reactive micro- and astrogliosis are prominent. We found that the spatial expression pattern of TJ markers claudin-5 and ZO-1 is not changed. Similarly, expression of laminin or α-dystroglycan were not altered. Expression of PDGFRβ was also unchanged, thus not giving a direct indication of pericyte dysfunction. There was, however, redistribution of AQP4 along the cell bodies and processes. This can point towards the alteration of ion-water homeostasis in PMD astrocytes in that could contribute to neurodegeneration.

In demyelinating leukodystrophies, the development of myelin is supposedly largely unaffected, but later in life loss of myelin (demyelination) occurs. Metachromatic leukodystrophy (MLD) is an autosomal recessive lysosomal sphingolipid storage disorder caused by *ARSA* mutations. These result in a deficiency of the enzyme arylsulfatase A (ASA), which is responsible for breaking down sulfatides. Sulfatides are major components of myelin. In MLD, accumulation of sulfatides is toxic and results in demyelination [24–27]. MRI shows bilateral signal changes in the periventricular and subcortical WM [28]. Contrast enhancement is observed in the cranial nerves of MLD patients, indicating leakage of the BBB at this site [29, 30]. Pathology reveals demyelination with sulfatide storage in glial cells, neurons and macrophages. We found that the expression pattern of claudin-5 and ZO-1 remains unchanged as well as that of PDGFRβ, indicating no obvious endothelial or pericyte dysfunction. Furthermore, the expression of laminin and α-dystroglycan seemed unchanged, indicating no direct disruption of the basal lamina. Expression of AQP4, however, was clearly redistributed, indicating an astrocytic dysfunction.

We also assessed the NVU in X-linked adrenoleukodystrophy (X-ALD), another demyelinating leukodystrophy [31]. X-ALD is caused by mutations in *ABCD1* encoding the ATP Binding Cassette Subfamily D Member 1 (ABCD1). ABCD1 transports very long‑chain fatty acid-CoA (VLCFA-CoA) into peroxisomes for degradation [32–35]. Dysfunction of ABCD1 results in impaired breakdown and accumulation of VLCFAs [36, 37]. In cerebral X-ALD, MRI signal changes most often progressively extend from the corpus callosum and occipital WM towards the frontal regions. Contrast enhancement is observed inside the leading edge of the lesion [38]. Pathology shows different concentric zones. The most outward zone is characterized by accruing of lipid-laden macrophages and lymphocytes, which supposedly facilitate the complete myelin breakdown observed in this zone [39]. The second zone is characterized by a strong inflammatory response with microglia activation and perivascular lymphocytic cuffings, together with large numbers of mononuclear cells [40]. This zone corresponds to the contrast enhancing areas [41]. The inner zone is characterized by a condensed net of glial fibrils and reactive astrocytes in the absence of myelin and oligodendrocytes. IgG and matrix metalloproteinases (MMPs) are increased in the cerebrospinal fluid (CSF) of X-ALD patients, indicating blood-CSF barrier and BBB breakdown [42, 43]. We found unchanged expression of claudin-5 in X-ALD, whereas ZO-1 is expressed outside the vasculature, indicating endothelial dysfunction. Expression of PDGFRβ and laminin appeared to be unchanged, not giving direct indication of pericyte or basal lamina disruption. α-dystroglycan however appeared to be downregulated in vessels with low UEA I expression. There was, additionally, clear redistribution of AQP4, indicating astrocyte dysfunction. 

*LARS2*-related leukodystrophy belongs to leukodystrophies with myelin vacuolization [44]. Diseases due to deficits in mitochondrial aminoacyl tRNA synthetases constitute a large proportion of the mitochondrial leukodystrophies [1]. MRI of *LARS2*-mutant patients shows diffusely abnormal cerebral WM and elevated lactate levels on the magnetic resonance spectroscopy. Pathology is characterized by involvement of oligodendrocytes and astrocytes with lack of myelin and deficient astrogliosis. No morphologic indications for vasculature involvement have been observed [44]. We found that expression of the TJ marker claudin-5 is not altered, whereas that of ZO-1 is dislocated outside the vasculature, indicating endothelial dysfunction. Expression of PDGFRβ was not changed. No clear alterations in α-dystroglycan expression were observed. Expression of laminin, interestingly, was observed outside the vasculature in non-perivascular astrocytes. In addition, there was clearly abnormal distribution of AQP4, also pointing to astrocytic dysfunction. 

Astrocytopathies are leukodystrophies caused by mutations in astrocyte-specific gene products or in which astrocytes significantly contribute to the disease mechanisms. Alexander disease (AxD) is the prototypic astrocytopathy. Different phenotypes are known, depending on the age at onset [45]. It shows contrast enhancement [46]. AxD is caused by dominant mutations in *GFAP,* causing accumulation of abnormally folded GFAP in astrocytes [47] resulting in the formation of Rosenthal fibers (RFs) [48]. RFs cause activation of cellular stress pathways [49], inhibition of proteasome activity [50] and changes in autophagy [51, 52]. RFs are mainly observed in astrocyte end feet in subependymal and subpial zones and around blood vessels, where they correlate with BBB dysfunction and contrast enhancement on MRI. Contrast enhancement on MRI is typically seen in the areas of highest RF density. We found unchanged expression of claudin-5, whereas ZO-1 expression is dislocated outside the endothelium, outside the UEA I positive RFs, pointing to endothelial dysfunction. We observed expression of UEA I in the GFAP positive RFs of the infantile-onset AxD patient. Expression of PDGFRβ was unchanged. Additionally, α-dystroglycan was downregulated in vessels with relatively low UEA I expression. Redistribution of AQP4 was observed, also outside the UEA I positive RFs. Since AxD is a prototype astrocytopathy, redistribution of AQP4 provides further evidence for astrocyte dysfunction beyond the formation of RFs.

Aicardi-Goutiéres syndrome (AGS) is another astrocytopathy [53]. It is caused by loss of function mutations in now nine known genes, namely *TREX1, RNASEH2A, RNASEH2B, RNASEH2C, SAMHD1, ADAR1,IFIH1/MDA, LSM11 and RNU7-1* [54]. These mutations result in upregulation of interferon alpha (IFN-α) [55−58]. Astrocytes are the main cell type that produce IFN-α in the CNS [59, 60]. MRI reveals calcifications in the basal ganglia and in the WM and WM signal changes in the frontotemporal regions. One study on 12 AGS patients found only one to show contrast enhancement [61]. Neuropathology shows lack of myelin with some infiltrating T-cells; calcifications due to increased IFN- α are located in the blood vessel tunica media [62]. We observed no alterations in the expression of claudin-5, yet there was redistribution of ZO-1, suggesting brain endothelial dysfunction. Furthermore, α-dystroglycan seemed downregulated in vessels with relatively low UEA I expression. The expression patterns of PDGFRβ and laminin were unchanged. The expression of AQP4 also showed clear redistribution, providing further proof for overall astrocyte dysfunction in AGS. 

Microgliopathies are leukodystrophies caused by mutations in microglia-specific gene products or in which microglia significantly contribute to the disease process. Adult-onset leukodystrophy with axonal spheroids and pigmented glia (ALSP) is caused by mutations in *CSF1R* encoding colony stimulating factor 1 receptor. MRI shows progressive WM signal changes and atrophy; no contrast enhancement is reported [63, 64]. Microscopy reveals WM vacuolization and myelin loss, and axonal spheroids. Reactive gliosis and scarce microglia activation are also seen, together with accumulation of lipid-laden macrophages and pigmented glia [65–67]. CSF1R is a tyrosine kinase receptor crucial for the functioning of microglia. Together with its ligands, CSF1 and IL-34, it regulates the production, differentiation, activation and chemotaxis of microglia and macrophages [68]. We found that the expression of claudin-5 and ZO-1 is unchanged. PDGFRβ and laminin expression is also unchanged, giving no direct indication for pericyte dysfunction and disruption of the basal lamina. However, expression of α-dystroglycan appeared downregulated in vessels with relatively low UEA I expression. Also, there was clear redistribution of AQP4 indicating that astrocytes could contribute to ALSP pathology. 

Leuko-vasculopathies are leukodystrophies characterized by involvement of the small blood vessels. Cathepsin A-related arteriopathy with strokes and leukoencephalopathy (CARASAL) is an adult-onset leuko-vasculopathy caused by a dominant mutation in *CTSA,* encoding cathepsin A (CathA). CathA is mostly expressed in the endothelium [69]. CathA is a lysosomal serine protease that stabilizes lysosomal activity and is also involved in ECM formation and stabilization [70, 71]. MRI shows diffuse WM signal changes with infarcts and microbleeds in the basal nuclei, brainstem and cerebellum. Neuropathology confirms atrophy of the cerebral WM and presence of small infarcts. At the arteriolar branches, the vessel walls display asymmetrical fibrous thickening and loss of vascular smooth muscle cells (VSMCs) with occlusion of the lumen. In some vessels there is also thickening of the basal lamina [72]. CathA degrades Endothelin-1 (ET-1), which is a small signaling peptide regulating vasoconstriction, but also multiple facets of oligodendrocyte progenitor proliferation and maturation [73, 74]. ET-1 is upregulated in CARASAL WM astrocytes. We found redistribution of ZO-1, while claudin-5 expression remained unchanged. This indicates that there is indeed endothelial dysfunction in CARASAL. No changes in the distribution of PDGFRβ were observed, however, we found reduced smooth muscle actin (SMA) expression [72], which is also a marker for pericytes. This points towards pericyte dysfunction in this disease. Laminin and α-dystroglycan expression was unchanged. Finally, there was redistribution of AQP4 in CARASAL, indicating astrocyte dysfunction.

## Discussion

Our immunohistochemistry results suggest that the NVU is abnormal in all the leukodystrophies investigated. All changes were consistent throughout the white matter examined. The pathology, however, resides at different levels of the NVU.

In AxD, AGS, CARASAL, *LARS2*-related leukodystrophy and X-ALD, we found expression of ZO-1 also outside of the NVU. Until now, little is known about TJ displacement. One study in X-ALD described co-localization of ZO-1 with IBA1-positive cells, which should be microglia [75]. Another study showed that peripheral leukocytes in multiple sclerosis (MS) patients express several TJ proteins [76]. In line with this, in an experimental autoimmune encephalomyelitis model of MS, endothelial cells (ECs) secrete claudin-5 via extracellular vesicles (EVs), which are then taken up by leukocytes [77]. One scenario is that TJs expressed by and/or taken up via EVs in leukocytes facilitate their transendothelial migration into the brain parenchyma using a “zipper mechanism” [78–80], which in turn promotes neurodegeneration. It could be speculated that ECs also secrete ZO-1 via EVs, which then are taken up by leukocytes and/or microglia. EVs secreted by ECs, however, can also be taken up by non-immune cells, including oligodendrocytes, astrocytes and pericytes [81, 82]. To date, the physiological relevance of these EVs secreted by ECs and the transfer of TJs via EVs remains an enigma. Interestingly, we did not observe abnormalities in the expression of claudin-5 in leukodystrophies, even in those with contrast enhancement. Claudin-5 dysfunction is considered one of the main drivers behind BBB leakage [83]. It should be noted however that our study was limited to conventional optic microscopy, not detecting alterations in proximity of individual proteins.

Pericyte or smooth muscle cell dysfunction can be envisioned in leukodystrophies due to microangiopathy, especially with blood vessel calcifications. Mutations in PDGFRβ and its ligand, PDGFB, are linked to the genetic disorder idiopathic basal ganglia calcification (IBGC). In the brain, PDGFB is mostly secreted by ECs [84]. Mice with partial activation of *Pdgfb* or *Pdgfrb* show brain calcifications and impaired BBB. This latter was linked to defective pericyte migration and proliferation. Endothelial PDGFB expression protects from brain calcifications and severity of calcifications correlates with the degree of BBB dysfunction and pericyte deficiency [85, 86]. These findings indicate a general role for pericytes in different diseases with brain calcifications. Unexpectedly, we found no PDGFRβ expression abnormalities in AGS, apparently excluding a pericyte pathogenic involvement. Calcification of the vessels in AGS, however, is mostly limited to arterioles, where the endothelium is lined by VSMCs and not pericytes, indicating that the calcification is not prominent in the capillary bed. Consistent with this, VSMCs show calcification upon stimulation with IFN-α [62]. No changes in the distribution of PDGFRβ were observed in CARASAL. We found however loss of SMA expression [72], which pericytes also need to contract properly, pointing towards pericyte dysfunction in this disease. Dysfunctional BBB with pericyte degeneration and astrocytic endfeet swelling has been generally reported in small vessel diseases together with vascular occlusions. These features alter the CBF decreasing the amount of nutrients entering the brain parenchyma and inducing BBB dysfunction [87–89]. Alterations in SMA expression, as in CARASAL, conceivably also alters the stiffness of the vessel wall and impair CBF [90, 91]. CBF is crucial for maintenance of constant energy supply to the brain [92]. If CBF is reduced, less glucose shuttles via the BECs into the brain parenchyma, disrupting energy metabolism and increasing levels of reactive oxygen species (ROS). Increased ROS cause mitochondrial dysfunction and, in turn, induce BBB damage. There is, however, a dual role for ROS at the NVU. Low concentrations of ROS are necessary for proper function of the NVU since they regulate vascular tone [93, 94]. Under pathological conditions, increased ROS levels disrupt the oxidant/anti-oxidant balance, resulting in oxidative stress. Oxidative stress plays a role in ALSP, as suggested by high levels of ceroid and iron in macrophages [95–97]. MRI imaging shows altered cerebral microvascular perfusion also in X-ALD [98]. This is compatible with alterations in CBF [99]. Indeed, oxidative stress plays a role in the pathology of X-ALD.

Redistribution of AQP4 in AxD, ALSP, PMD, *LARS2*-related leukodystrophy, X-ALD and CARASAL strongly points towards an astrocytic dysfunction. AQP4 redistribution is a common phenomenon in glial tumors, including glioblastoma [100, 101]. Here, increased levels of AQP4 and its redistribution facilitate edema formation, thereby altering ion-water homeostasis. AQP4 redistribution is also observed leukodystrophies caused by adult-onset small vessels diseases other than CARASAL, including CADASIL and CARASIL. In these diseases, AQP4 redistribution is associated with retraction of astrocyte end feet from the glial basal lamina [102, 103]. In the context of leukodystrophies, astrocytic morphology and function have only been studied in a few astrocytopathies, but no data about end feet localization are available from systematic ultrastructural studies. One exception is megalencephalic leukoencephalopathy with subcortical cysts, a leukodystrophy caused by MLC1 or GlialCAM mutations encoding for components of the dystrophin associated glycoprotein complex, coupled to AQP4 water channels and Kir4.1 K + channels [104, 105]. Here, AQP4 redistribution does not associate with astrocyte end feet retraction, but rather with astrocyte end feet vacuolization, compatible with a defect of ion-and-water homeostasis at the endfeet. In addition, the possibility of a final common involvement of astrocyte end feet across leukodystrophies has also not been investigated. However, we found downregulation of α-dystroglycan expression in blood vessels with relatively low UEA I expression in AxD, ALSP, X-ALD and AGS, which could hint to astrocyte dysfunction by retraction of the astrocyte endfeet. As mentioned before, however, our study is limited to optic microscopy. For demonstrating astrocyte endfeet retraction, electron microscopy is needed. In AxD, astrocytes accumulate GFAP, especially at end feet, which leads to formation of RFs, proteasome inhibition and increased nitric oxide and chemokine production. Astrocyte endfeet pathology could mechanically contribute to NVU dysfunction. Also, cell stress pathways are activated, oxidative stress is increased and astrocyte physiologic functions are overall compromised [106]. In X-ALD, accumulating VLCFAs in astrocytes are speculated to cause mitochondrial dysfunction [107]. Astrocytes show increased expression of heat-shock proteins, which points towards increased cellular stress [108]. Disease mechanisms in *LARS2*-related leukodystrophy are still elusive. Nonetheless, AQP4 redistribution points to astrocyte dysfunction. The involvement of mitochondrial leucyl tRNA synthetase in mitochondrial translation could suggest an astrocytic dysfunction due to mitochondrial failure. In CARASAL, ET-1 upregulation could also influence AQP4 distribution. In a rodent model of ischemic stroke, ET-1 overexpression results in upregulation of AQP4 at astrocyte endfeet [109]. The cells that drive disease pathology in ALSP are microglia. Interplay between microglia and astrocytes could change the astrocytes into a reactive state [109, 110]. In all these diseases, AQP4 redistribution away from astrocyte end feet suggests that at least the function of ion-and-water regulation at the NVU is generally compromised.

Neuroinflammation via cytokine production may play a prominent role in some, but certainly not all leukodystrophies. In PMD, increased astrogliosis and microgliosis characterize the brain pathology [111]. A mouse model with increased *PLP1* expression shows massive microglia activation and increased tumor necrosis factor alpha (TNFα) and interleukin-6 (IL-6) levels [112]. Similarly, in MLD, loading human monocytes in vitro with sulfatides results in a significant increase of the cytokines TNFα, IL-1β, IL-8 and IL-6 [113]. TNFα and IL-6 secreted by astrocytes and microglia regulate immune cell infiltration through the BBB [114–117]. IL-6 can also be secreted by pericytes stimulated with TNFα. This further induces microglia and astrocyte activation [118–120]. IL-6 overproduction directly leads to neurodegeneration [114].

Neuroinflammation in ALSP involves a different cytokine profile. In the CNS, CSF1 is produced by astrocytes, oligodendrocytes, and microglia, whereas IL-34 is expressed by neurons. These ligands have separate roles in maintaining microglia pool in the WM and GM [121]. A recent study showed amyloid-β depositions in blood vessels of ALSP patients, indicating cerebral amyloid angiopathy. This study proposed that the driving force behind ALSP pathology is systemic inflammation [122]. Neuroinflammation could also play a role in AxD that features perivascular and intraparenchymal T-lymphocytic infiltrates [123–126]. Downstream effectors of the NF-κB inflammatory signaling pathway are significantly upregulated in this disease. AxD is characterized by activated microglia/macrophages and T cells [124]. This could theoretically contribute to BBB disruption. In AGS, there is co-localization of IFN-α and C-X-C motif ligand (CXCL) 10 with GFAP [127]. CXCL10 is an important recruiter of leukocytes and is specifically secreted by astrocytes, thereby promoting further neuroinflammation and neurodegeneration [128].

Neuroinflammation via matrix metalloproteinases (MMPs) could play a role in X-ALD. In this leukodystrophy, increased MMP9 expression in the perilesional WM was found in microglia, astrocytes and endothelial cells [75]. Previous studies showed that MMP9 upregulation can be triggered by TNFα and IL-1β [129], which are also found to be upregulated in X-ALD, and that increased levels of MMP9 are associated with early BBB disruption. Furthermore, infiltrated leukocytes and activated microglia are observed in the X-ALD WM. It could be speculated that, in analogy to other demyelinating neurodegenerative diseases as multiple sclerosis, infiltration of leukocytes has detrimental effects on disease progression as the neuroinflammatory cascade leads to further neurodegeneration also through BBB breakdown.

The participation of microglia to NVU dysfunction in leukodystrophies is still unknown. In many of these diseases, microglia are generally quite asthenic, underestimated or simply not studied. The only notable exceptions are X-ALD and PMD, in which microgliosis is prominent, and ALSP, due to genetic defects in microglia proliferation and activation. Further studies on microglia in leukodystrophies are needed [2].

## Conclusions

Taken altogether, others’ and our findings suggest an NVU involvement in several leukodystrophies. Especially AQP4 redistribution points towards a reactive astrocytes in not only astrocytopathies, but also in leukodystrophies where reactive astrogliosis occurs. Notably, different leukodystrophies without contrast enhancement on MRI also show abnormalities at the brain vascular unit. Some of these exhibit neuroinflammation. Vessels calcification and consequent occlusion support alterations in CBF that can influence BBB functioning in a bidirectional manner. Some leukodystrophies exhibit oxidative stress with ROS release, which also influence BBB functioning. Involvement of PDGFRβ signaling and pericyte function in leukodystrophies with calcifications should be looked into. Concluding, orchestration of the NVU in leukodystrophies is affected at different levels, sometimes multiple levels simultaneously: altered cerebral blood-flow, intrinsic disruption of the BECs and disruption of glial cells, in particular astrocytes.

## Data Availability

The materials and datasets used and/or analyzed during the current study are available from the corresponding author upon reasonable request.

## Abbreviations

ABCD1: ATP Binding Cassette Subfamily D Member 1
ADAR1: Gene encoding for adenosine deaminase
AGS: Aicardi-Goutières syndrome
AJ: Adherens junction
ALSP: Adult-onset leukodystrophy with spheroids and pigmented glia
AQP4: Aquaporin-4
ARSA: Gene encoding for Arylsulfatase A
ASA: Arylsulfatase A
ATP: Adenosine triphosphate
AxD: Alexander disease
BBB: Blood–brain barrier
BEC: Brain endothelial cell
CARASAL: Cathepsin A-related arteriopathy with strokes and leukoencephalopathy
CathA: Cathepsin A
CBF: Cerebral blood flow
CNS: Central nervous system
CSF: Cerebrospinal fluid
CSF1: Colony stimulating factor 1
CSF1R: Colony stimulating factor 1 receptor
CXCL: C-X-C motif ligand
ECM: Extracellular matrix
EC: Endothelial cell
ET-1: Endothelin-1
EV: Extracellular vesicle
GFAP: Glial fibrillary acidic protein
GM: Gray matter
IBGC: Idiopathic basal ganglia calcification
IFIH1/MDA: Interferon-induced helicase C domain-containing protein 1
IFN-α: Interferon alpha
IL-1β: Interleukin-1 beta:
IL-34: Interleukin-34
IL-6: Interleukin-6
IL-8: Interleukin-8
LARS2: Gene encoding for aminoacyl tRNA synthetase
MLD: Metachromatic leukodystrophy
MMP: Matrix metalloproteinase
MRI: Magnetic resonance imaging
Nf-κB: Nuclear factor kappa-light-chain-enhancer of activated B cells
NVU: Neurovascular unit
OAPs: Orthogonal arrays of intramembranous particles
PBS: Phosphate buffered saline
PDGFRβ: Platelet-derived growth factor receptor beta
PLP1: Proteolipid protein 1
PMD: Pelizaeus-Merzbacher disease
RF: Rosenthal fibers
RNASEH2A: Ribonuclease H2 Subunit A
RNASEH2B: Ribonuclease H2 Subunit B
RNASEH2C: Ribonuclease H2 Subunit C
ROS: Reactive oxygen species
SAMHD1: SAM domain and HD domain-containing protein 1
SMA: Smooth muscle actin
TJ: Tight junction
TNFα: Tumor necrosis factor
TREX1: Three Prime Repair Exonuclease 1
UEA I: Ulex europaeus agglutinin I
VLCFA-CoA: Very long‑chain fatty acid-CoA
VSMC: Vascular smooth muscle cell
WM: White matter
X-ALD: X-linked adrenoleukodystrophy
ZO-1: Zona occludens 1
